# LINC00675 activates androgen receptor axis signaling pathway to promote castration-resistant prostate cancer progression

**DOI:** 10.1038/s41419-020-02856-5

**Published:** 2020-08-15

**Authors:** Mengfei Yao, Xiaolei Shi, Yue Li, Yutian Xiao, William Butler, Yongqiang Huang, Leilei Du, Tianqi Wu, Xiaojie Bian, Guohai Shi, Dingwei Ye, Guohui Fu, Jianhua Wang, Shancheng Ren

**Affiliations:** 1 Cancer Institute, Fudan University Shanghai Cancer Center, Shanghai Urological Cancer Institute, Department of Oncology, Shanghai Medical College, Fudan University, Shanghai, China; 2 Department of Urology, Changhai Hospital, Navy Medical University, Shanghai, China; 3 Department of Pathology Center, Shanghai General Hospital/Faculty of Basic Medicine, Shanghai Jiao Tong University School of Medicine, Shanghai, China; 4 Depatment of Pathology, Duke University School of Medicine, Durham, NC USA; 5 Department of Urology, Fudan University Shanghai Cancer Center, Department of Oncology, Shanghai Medical College, Fudan University, Shanghai, China

**Keywords:** Tumour biomarkers, Prostate cancer

## Abstract

The development of prostate cancer (PCa) from androgen-deprivation therapy (ADT) sensitive to castration resistant (CRPC) seriously impacts life quality and survival of PCa patients. Emerging evidence shows that long noncoding RNAs (lncRNAs) play vital roles in cancer initiation and progression. However, the inherited mechanisms of how lncRNAs participate in PCa progression and treatment resistance remain unclear. Here, we found that a long noncoding RNA LINC00675 was upregulated in androgen-insensitive PCa cell lines and CRPC patients, which promoted PCa progression both in vitro and in vivo. Knockdown of LINC00675 markedly suppressed tumor formation and attenuated enzalutamide resistance of PCa cells. Mechanistically, LINC00675 could directly modulate androgen receptor’s (AR) interaction with mouse double minute-2 (MDM2) and block AR’s ubiquitination by binding to it. Meanwhile, LINC00675 could bind to GATA2 mRNA and stabilize its expression level, in which GATA2 could act as a co-activator in the AR signaling pathway. Notably, we treated subcutaneous xenografts models with enzalutamide and antisense oligonucleotides (ASO) targeting LINC00675 in vivo and found that targeting LINC00675 would benefit androgen-deprivation-insensitive models. Our findings disclose that the LINC00675/MDM2/GATA2/AR signaling axis is a potential therapeutic target for CRPC patients.

## Introduction

Prostate cancer (PCa) is the most commonly diagnosed male malignancy and second frequent cause of mortality^1^. Radical surgery and radiation therapy are the standard treatments for localized PCa. Androgen-deprivation therapy (ADT) is an effective treatment for locally advanced, biochemically recurrent or metastatic PCa^2^. However, most PCa patients that are initially sensitive to ADT will develop lethal CRPC inevitably. Dysregulation of the androgen receptor (AR) signal pathway is critical for PCa development and progression into CRPC^3^. Most PCa that relapses during ADT remains dependent on AR activity, in which events such as AR amplification and stabilization of mRNA and protein^4^, sensitization to low levels of androgen or steroid analog^5^, intratumoral de novo steroidogenesis^6^, or AR splice variant production^7^ may occur. The underlying mechanism and potential target that drive PCa into CRPC need to be illustrated so that novel therapeutics can be developed to be used in combination with ADT, which may be more effective in prolonging survival.

The ubiquitin–proteasome system plays vital roles in several biological processes, and protein ubiquitylation requires three processes, including the ubiquitin-activating enzyme (E1), ubiquitin-conjugating enzymes (E2s), and ubiquitin ligases (E3s). Mouse double minute-2 (MDM2), as a ring finger-containing E3, has been found to be involved in the regulation of AR ubiquitylation and degradation, by directly binding to the N-terminal and DBD of AR protein.

Long noncoding RNAs (lncRNAs) are known as a group of transcripts with lengths exceeding 200 nucleotides and not translated into protein^8^. LncRNAs used as diagnostic biomarkers have received sustained attention because they are expressed in a more tissue-specific manner than protein-coding genes^9^. Emerging evidence shows that lncRNAs play vital roles in cancer initiation and progression^10^. In PCa, lncRNAs such as PCA3 and SChLAP1 were reported as diagnostic and prognostic biomarkers^11,12^. PCa-associated lncRNAs were reported to participate in tumor progression^13^, castration resistance^14,15^, and metastasis^16^ via diverse mechanisms, including recruitment of the chromatin-modifying complex, chromatin remodeling, or transcriptional co-regulation, and by interacting with RNAs or proteins at post-transcriptional levels. Our previous studies revealed that lncRNAs were implicated in PCa progression and AR signaling pathway^13,17^. Li et al. found that LINC00675 was highly expressed in pancreatic ductal adenocarcinoma compared with adjacent normal tissue, and was positively correlated to lymph-node metastasis and poor survival^18^. Finally, LINC00675 was reported to be involved in cell proliferation, metastasis, and invasion in gastric cancer, colorectal cancer, and cervical cancer^19–21^. To date, the role of LINC00675 in PCa still remains unsettled.

In this study, we discovered that LINC00675 is significantly enriched in androgen-insensitive PCa cell lines and tissue specimens of CRPC patients. LINC00675 binds to AR protein and blocks its ubiquitination under androgen-deprivation conditions. Moreover, we demonstrated that LINC00675 could bind to GATA2 mRNA and stabilize its expression level, and then GATA2 can act as a co-activator in AR signaling in the nucleus. Our results suggest that the LINC00675/MDM2/GATA2/AR signaling axis contributes to the castration resistance and progression of PCa and is a promising therapeutic target.

## Materials and methods

### Cell culture

LNCaP-SF and its relative control LNCaP-JP human PCa cells were provided and cultured as recommended by Professor Atsushi Mizokami (Kanazawa University, Kanazawa, Japan). LNCaP-SF was constructed from the androgen-dependent cell line LNCaP-JP. The culture medium was replaced every 2–3 days with 5% charcoal-stripped fetal bovine serum (FBS; without steroids), then LNCaP-JP cells were cultured in androgen-deficient medium for over 6 months to construct stable LNCaP-SF cell line. LNCaP-SF was cultured in DMEM medium containing 10% charcoal-stripped fetal bovine serum, while its control cell line LNCaP-JP was cultured in DMEM medium containing 10% FBS. LNCaP-Abl cell was kindly provided by professor Jun Qin (Chinese Academy of Sciences, Shanghai, China) and cultured in RPMI-1640 medium containing 10% FBS. Its control cell line LNCaP was cultured as LNCaP-JP. LNCaP-C4–2b was obtained from American Type Culture Collection (ATCC) and cultured in T-medium containing 10% FBS. T-medium was prepared according to standard protocols. Enzalutamide (MDV3100)-resistant C4–2b (LNCaP-C4–2b-ENZ) cell was kindly provided by Dr. Allen Gao (University of California, CA, USA) and maintained in DMEM containing 20 μM enzalutamide and 10% FBS. BSA (A8022), triiodo-L-thyronine (T2877), transferrin (T4382), D-biotin (47868), and adenine (A3159) were purchased from Sigma-Aldrich. Insulin (12585–014) was purchased from Gibco.

The cells were all cultured in a 37 °C water-saturated 5% CO_2_ atmosphere.

### Plasmids

The plasmids used to knock down LINC00675, and its vector GV248 were purchased from Genechem, Inc (Shanghai, China). Sequences of the shRNAs used to knock down LINC00675 were as follows: (1) forward: 5′- CUCCGAUCUUUCAUGGUAU-3′; reverse: 5′-AUACCAUGAAAGAUCGGAG-3′; (2) forward: 5′-CAUAGGCUAACUGGAUAGA-3′; reverse: 5′-UCUAUCCAGUUAGCCUAUG-3′; (3) forward: 5′-CAGUGGCUCAUAUUGAGGU-3′; reverse: 5′-ACCUCAAUAUGAGCCACUG-3′. The plasmids used to overexpress AR, and its vector LvEKP01 were purchased from Era Biotech, Inc (Shanghai, China). The Flag-tagged pcDNA3.1-AR, pcDNA3.1-AR-truncated, and LvEKP01-LINC00675 were self-constructed. Full-length LINC00675, AR, and truncated AR (AR-NT, AR-DBD, AR-LBD, AR-NT-DBD, AR-DBD-LBD) were amplified by PCR and subcloned into the relative sites of the LvEKP01 vector or pcDNA3.1 vector.

### Generation of stable cell lines

293T cell was used to generate lentiviruses and seeded in 10-cm culture dishes before transfection. For the knockdown experiment, 293T cells were transfected with shLINC00675 plasmids or its vector with the packaging plasmids psPAX2 and pMD2.0 G using Lipofectamine 3000 (L3000015, Invitrogen, MA, USA). For the overexpression experiment, 293T cells were transfected with pcDNA3.1-AR, pcDNA3.1-AR-truncated, LvEKP01-LINC00675, and its relative vectors with the packaging plasmids pGag-pol, pREV, and pVSVG. Supernatant was collected 48 h after transfection, and filtered through 0.45-μm sterilization filters. Then 1 ml of lentiviruses, 1 ml of culture medium, and 2 μl of polybrene (8 μg/μl, TR-1003, Sigma-Aldrich, MO, USA) were applied to the cells. Culture medium was replaced after 8–12 h, and the stable cells were selected with puromycin (1 μg/μl, Sigma-Aldrich, MO, USA) or flow cytometry.

### RNA sequencing

The total RNA was extracted using TRIzol^TM^ Reagent, and RNA quality was checked using an Agilent 2100 Bioanalyzer (Agilent Technologies, Santa Clara, CA, USA). Then the total RNA was purified with the RNA Clean XP Kit (A63987, Beckman Coulter, Kraemer Boulevard Brea, CA, USA) and DNase set (79254, QIAGEN, Germany). Afterward, 1000 ng of RNA was used as templates to synthesize cDNA with VAHTS^®^ mRNA-seq V2 Library Prep Kit for Illumina (NR603, Vazyme, Nanjing, China). Finally, cDNA was amplified by PCR, quality checked using Bioanalyzer (Agilent Technologies, Santa Clara, CA, USA), and sequenced using Illumina HiSeq 4000 platform (Illumina, San Diego, CA, USA).

### RNA pulldown

Full-length LINC00675 was amplified by PCR. The primer sequences are as follows: LINC00675-sense-F: 5′- TAATACGACTCACTATAGGGAGAGTGGCTCCAAGAAGCGCCAG-3′; LINC00675-sense-R: 5′-TTACATAAAAACAAAGGCCAAATCT-3′. Then LINC00675 RNA was biotinylated using Biotin RNA Labeling Mix Kit (11685597910, Roche, Basel, Switzerland) and in vitro transcribed using T7 polymerase (M0251, NEB, MA, USA). After this, 3 μg of biotinylated LINC00675 RNA was incubated with 1 mg of total cell lysates to bind to the protein of interest at 4 °C overnight. Then, 30 μl Dynabeads^TM^ M-280 Streptavidin (11205D, Invitrogen, MA, USA) was added to the RNA–protein complex and incubated with the complex at room temperature for 2 h. After washes, the pull-down complexes were denatured and detected by western blot.

### Cell-viability assay

Cell Counting Kit-8 (CCK-8) (Dojindo, Japan) was used to assess the long-term cell survival according to the manufacturer’s instructions. Cell suspensions were seeded at 5 × 10^3^ cells per well in 96-well culture plates. Cell viability was determined by CCK-8 at a final concentration of 10% to each well, and the absorbance of the samples was measured at 450 nm using an Epoch^TM^ Microplate Spectrophotometer (Biotek, VT, USA) every 24 h for 5 days. The proliferation curves were plotted using the absorbance.

### Mouse xenograft experiments

Male BALB/c nude mice, 4–5 weeks old, were purchased from the Department of Laboratory Animal Science in Shanghai Jiao Tong University. To evaluate the effect of LINC00675 knockdown on tumor growth in vivo, mice were subcutaneously inoculated with 1 × 10^7^ LNCaP-C4–2b^shGFP^和LNCaP-C4–2b^shLINC00675^ cells. Tumor growth was monitored every 3 days after a week post inoculation. Tumor volume was calculated according to the formula: volume = length × width^2^ × 0.5. After 4 weeks, the mice were euthanized, and tumors were harvested, weighed, and subjected to IHC and western blot analyses.

LNCaP-C4–2b-ENZ cells (1 × 10^7^) were mixed with Matrigel (356234, Becton Dickinson, NJ, USA) at a ratio of 1:1 and subcutaneously injected into the 6-week-old male BALB/c nude mice. When the tumors were touchable, the mice were treated with enzalutamide and/or antisense oligonucleotides (ASO). In all, 10 mg/kg enzalutamide was intraperitoneal injected every 2 days. The in vivo-jetPEI^TM^ DNA & siRNA Delivery Kit (201–10 G, Polyplus-transfection, France) was used to knock down LINC00675 in vivo. In total, 20 μg of ASO and 2.4 μl in vivo-jetPEI reagent were, respectively, dissolved in 12.5 μl 10% glucose solution, then mixed and diluted into 50 μl. After incubation at room temperature for 15 min, the complex was injected into the tumors at a frequency of twice a week. Formalin-fixed paraffin-embedded sections of subcutaneous tumors were subjected to IHC.

### Immunohistochemistry (IHC)

Formalin-fixed paraffin-embedded sections from specimens were baked at 60 °C for 4 h, deparaffinized by three times of 10-min extraction in 100% xylene and rehydrated in a series of graded alcohol (100%, 95%, 80%, 70%, 5 min each). After washing with phosphate buffered saline (PBS), sections were transferred to boiling sodium citrate buffer (10 mM, pH = 6.0) to retrieve antigen for 30 min and pre-treated with 3% H_2_O_2_ for 10 min. Tissue sections were blocked with 5% goat serum in PBS for 30 min at room temperature, followed by primary antibody incubation at 4 °C overnight. After washed with PBS for three times, 5 min each, sections were incubated with relative secondary antibody for 30 min at room temperature. Then, the 3,3’-diaminobenzidine (DAB) kit was used for detection. The quantitative results for IHC images were calculated by ImageJ software.

The primary antibodies are as follows: Ki67 (ab15580, Abcam, MA, USA) and AR (74272, Abcam, MA, USA) were commercially purchased.

### Western blots and antibodies

Western blots were performed according to standard methods. After relative treatment, cells were washed three times with pre-chilled PBS and lysed for 30 min on ice with RIPA buffer (89900, Thermo Scientific, MA, USA) containing protease and phosphatase-inhibitor mixture (P1048, Beyotime Biotechnology, Shanghai, China). Lysates were centrifuged at 12,000 rpm for 15 min at 4 °C. Protein concentration of the supernatants was determined using a BCA Protein Assay Kit (23225, Thermo Scientific, MA, USA). The protein fractions were resuspended in loading buffer and denatured at 100 °C for 10 min. Equal amount of proteins was resolved by 10% SDS polyacrylamide gels and transferred to PVDF membranes (IPVH00010, Millipore, MA, USA). Membranes were blocked in 5% fat-free milk in TBST buffer (0.1% Tween-20) for 1 h at room temperature and then incubated with specific antibodies for different western blot analyses at 4 °C overnight.

The antibodies used in the assays are listed in the Supplementary Materials.

### Statistics

Statistical analyses were conducted using SPSS19.0 software, and graphs were generated using GraphPad Prism 6.0 software. All in vitro experiments were performed in biological triplicate. Data were presented as the mean ± SD. Continuous variables were compared by Student’s *t* test for variables with normal distribution, or the Mann–Whitney *U* test for variables without normal distribution; categorical variables were compared by Pearson’s Chi-square test or Fisher’s exact test. Two-sided *P*-values <0.05 were considered statistically significant.

## Results

### LINC00675 is highly expressed in CRPC cells and tumor tissues

**Fig. 1:**
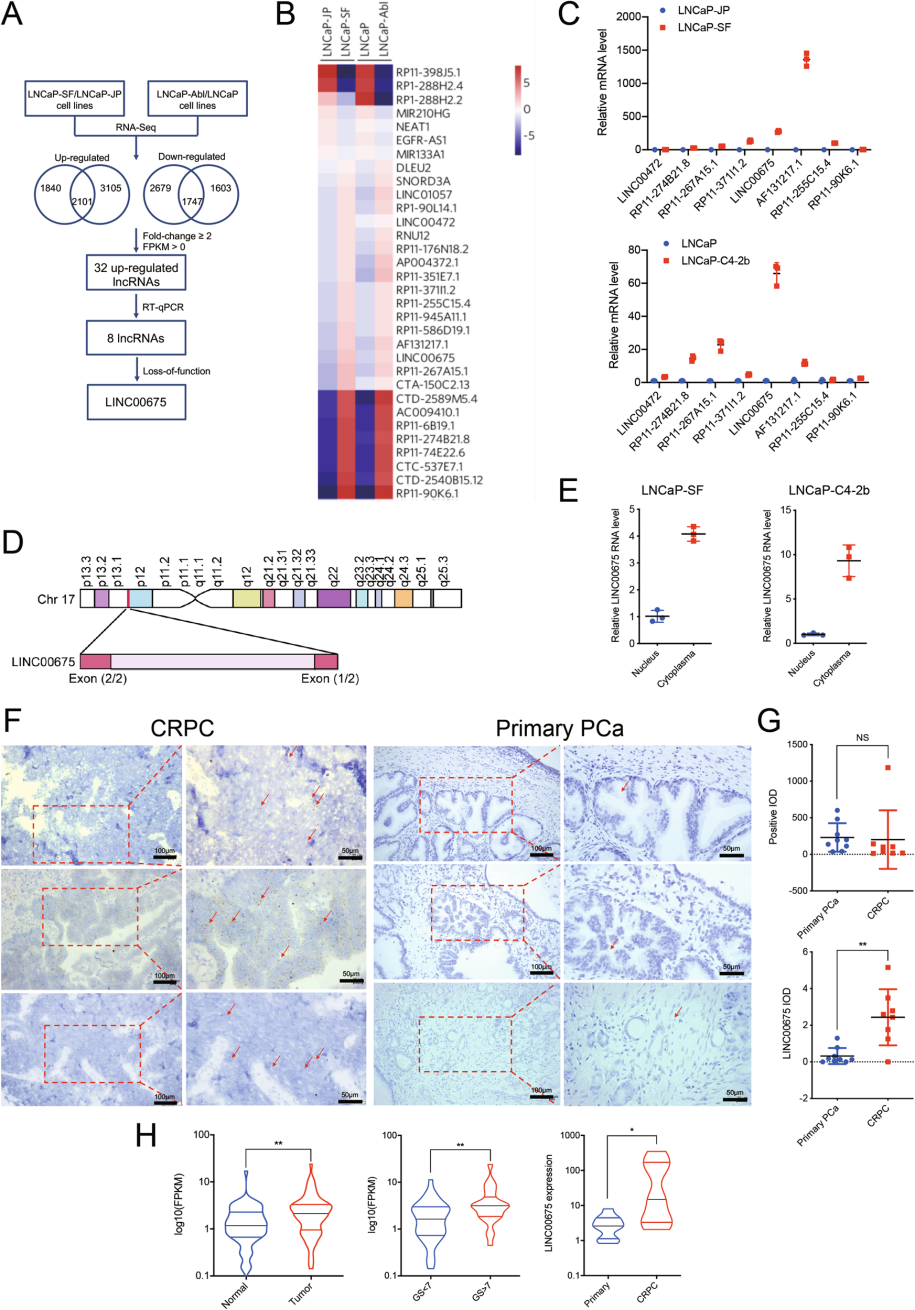
Identification of LINC00675 in androgen-sensitive/insensitive prostate cancer (PCa) cells. **a** Schematic diagram of LINC00675 screening. **b** Heatmaps of 32 differentially expressed lncRNAs via RT-qPCR. LINC00675 was highly expressed in androgen-independent PCa cell lines. **c** Expression levels of highly expressed lncRNAs in androgen-independent PCa and control cell lines via RT-qPCR. *n* = 3. Data are represented as mean ± SD. **d** Schematic annotation of LINC00675 genomic locus on intergenic region of chromosome 17. **e** Nuclear and cytoplasmic RNA isolation assay showed that LINC00675 is highly expressed in the cytoplasm rather than nucleus. *n* = 3. Data are represented as mean ± SD. **f**, **g** Expressions of LINC00675 in primary PCa and castration-resistant prostate cancer (CRPC) patients using RNA-scope. Representative images of CRPC (**f**, left, *n* = 8) and primary PCa (**f**, right, *n* = 9) tissues samples. Quantitative analysis of LINC00675 IOD (**g**, upper) compared with positive control (**g**, lower). Scale bar: left column, 100 μm; right column, 50 μm. IOD integral optical density. NS not significant. Data are represented as mean ± SD. **h** LINC00675 expression levels in specimens of PCa patients between (left) tumor tissue samples and normal control, *n* = 66; (middle) Gleason score <7 (*n* = 41) and >7 (*n* = 24); (right) primary PCa (*n* = 10) and CRPC (*n* = 10) via RT-qPCR. Data are represented as median ± quartiles. GS Gleason score, NS not significant. *P < 0.05; **P < 0.01.

To explore the molecular mechanism by which PCa develops from androgen-dependent to androgen-independent status, we chose two androgen-independent cell lines (LNCaP-SF & LNCaP-Abl) and respective androgen-dependent parental cell lines (LNCaP-JP & LNCaP) to assay lncRNA expression profiles using RNA sequencing (Fig. 1a). Fold change ≥2 screening between androgen-independent cell lines and their relative controls and FPKM > 0 in any cell lines identified 32 lncRNAs among upregulated genes (Fig. 1b). Afterward, we used RT-qPCR to identify eight upregulated lncRNAs in two different androgen-independent cell lines LNCaP-SF and LNCaP-C4–2b (Fig. 1c). The eight selected lncRNAs were subjected to loss-of-function analyses in androgen-independent PCa cells using RNAi. Notably, RNAi-mediated silencing of LINC00675 (Ensembl: ENSG00000263429) greatly suppressed cell growth compared with the effects of the other six lncRNAs (LINC00472 excluded, Supplementary Fig. S1A). Based on this observation, we next observed that LINC00675 is located on chromosome 17 in humans and composed of two exons with a full length of 1530 nt (Fig. 1d). The noncoding nature of LINC00675 was confirmed by coding-potential analysis (Supplementary Fig. S1B). Using RT-qPCR and FISH, we confirmed that LINC00675 expression was higher in the cytoplasma than nucleus, and higher in enzalutamide-resistant LNCaP-C4–2b-ENZ than LNCaP-C4–2b (Fig. 1e; Supplementary Fig. S1C). To identify the correlation of LINC00675 expression with PCa patients, RNA-scope was performed using nine primary PCa tissues and eight CRPC tissues. As shown in Fig. 1f, LINC00675 expression was higher in CRPC patients than primary PCa patients. The quantification assay confirmed the observation (Fig. 1g). Meanwhile, using Ren’s RNA-seq data^22^, we also found that LINC00675 expression was markedly increased in tissues of PCa patients compared with tissues of normal prostate (Fig. 1h). A similar result was also noted in PCa tissues between Gleason score >7 and Gleason score <7 groups, and PCa tissues between primary cancer and CRPC (Fig. 1h; Supplementary Fig. S1D). Taken together, these data suggest that LINC00675 expression is associated with CRPC and PCa progression.

### LINC00675 promotes PCa progression

**Fig. 2:**
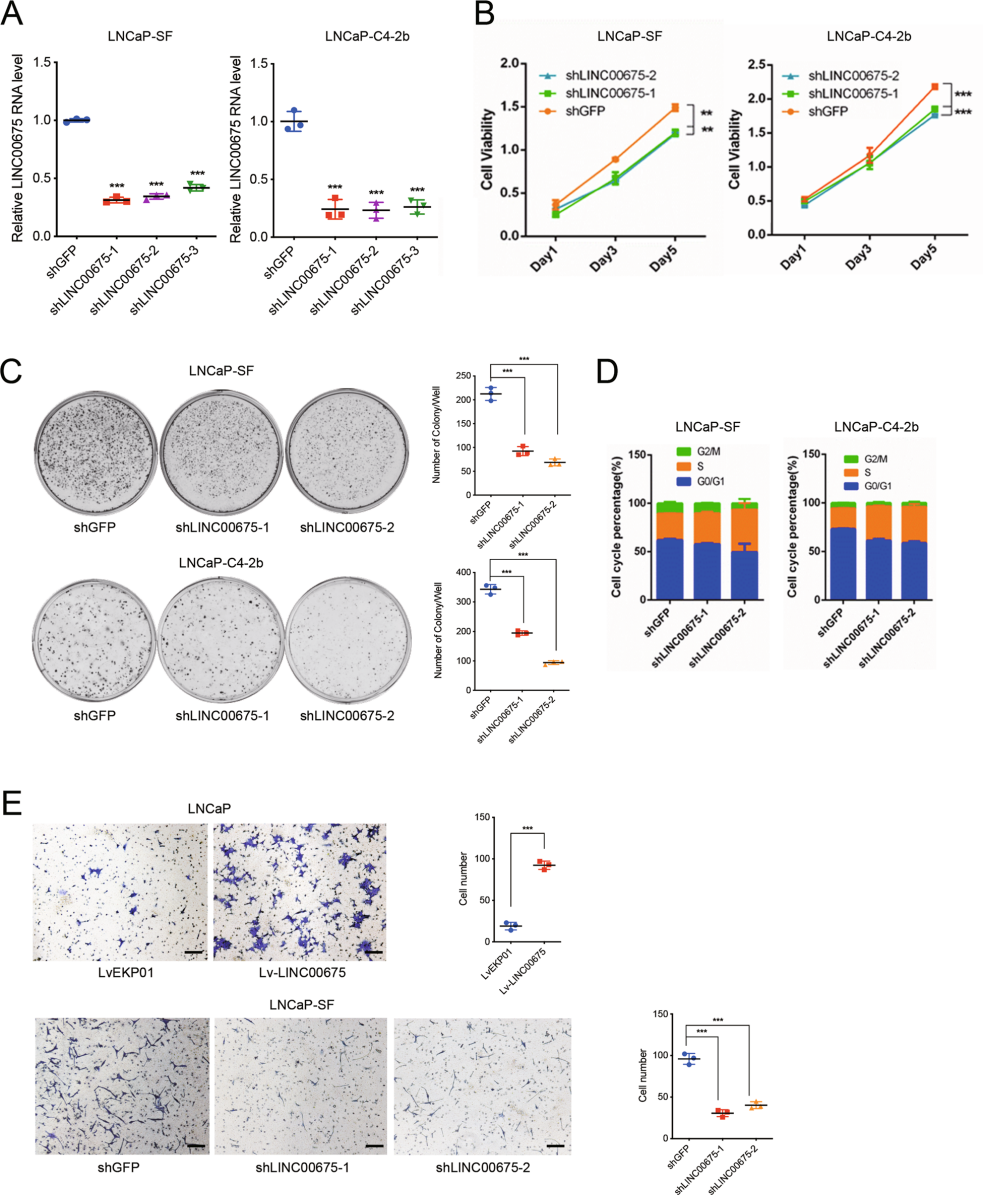
LINC00675 promotes prostate cancer (PCa) development. **a** Knockdown efficiency of LINC00675 in LNCaP-SF and LNCaP-C4-2b via lentivirus transfection. *n* = 3. **b** Cell viability of LNCaP-SF and LNCaP-C4-2b with LINC00675 knockdown were determined by CCK-8 kit. **c** Colony-formation assay of LNCaP-SF and LNCaP-C4-2b with LINC00675 knockdown. Left: representative images of colony formation; right: quantitative analysis of colonies numbers per well. *n* = 3. **d** Cell cycle changes of LNCaP-SF and LNCaP-C4-2b with LINC00675 knockdown were determined by flow cytometry. **e** Migration assay of LNCaP with LINC00675 overexpression and LNCaP-SF with LINC00675 knockdown. Left: representative images of migrated cells; right: quantitative analysis of cell numbers. Scale bar: 20 μm. *n* = 3. Data are represented as mean ± SD. **P < 0.01; ***P < 0.001.

To explore the potential function of LINC00675 on PCa progression, we constructed three short-hairpin RNAs (shRNAs) to inhibit LINC00675 expression in LNCaP-SF and LNCaP-C4–2b (Fig. 2a). Knockdowns of LINC00675 markedly attenuated cell viability (Fig. 2b), and suppressed colony-formation ability of LNCaP-SF and LNCaP-C4-2b cells (Fig. 2c) compared with the respective controls. Cell cycle analysis using flow cytometry showed that LINC00675 depletion in LNCaP-SF and LNCaP-C4-2b cells resulted in cell growth arrest in the S phase (Fig. 2d). Furthermore, using RNAi-mediated silencing of LINC00675, we noted that cell viability was greatly attenuated in enzalutamide-resistant cell line LNCaP-C4-2b-ENZ under the treatment of 20 μM enzalutamide (Supplementary Fig. S2A). Migration assay showed that gain of LINC00675 in LNCaP promoted cell-migration ability and knockdown of LINC00675 in LNCaP-SF suppressed cell migration ability (Fig. 2e). Using western blot assay, we screened epithelial–mesenchymal transition (EMT)-related markers. Overexpression of LINC00675 in LNCaP showed downregulation of E-cadherin expression, upregulation of N-cadherin and vimentin expression, whereas knockdown of LINC00675 in LNCaP-C4-2b resulted in the opposite results (Supplementary Fig. S2B). Finally, subcutaneous xenografts generated by injecting LINC0067-depleted LNCaP-C4-2b or control cells were used to assay in vivo effects (Supplementary Fig. S2C). As shown in Supplementary Fig. S2D, knockdown of LINC00675 significantly repressed tumor growth in volume and weight compared with the control. Western blot and immunohistochemistry assays of mice tumor specimens showed decreased AR and Ki67 expression in LINC00675-depleted tumors (Supplementary Fig. S2E). Taken together, the results suggest that LINC00675 is an oncogenic lncRNA, which is associated with PCa progression by modulating AR expression.

### LINC00675 stabilizes AR protein by blocking its ubiquitination

**Fig. 3:**
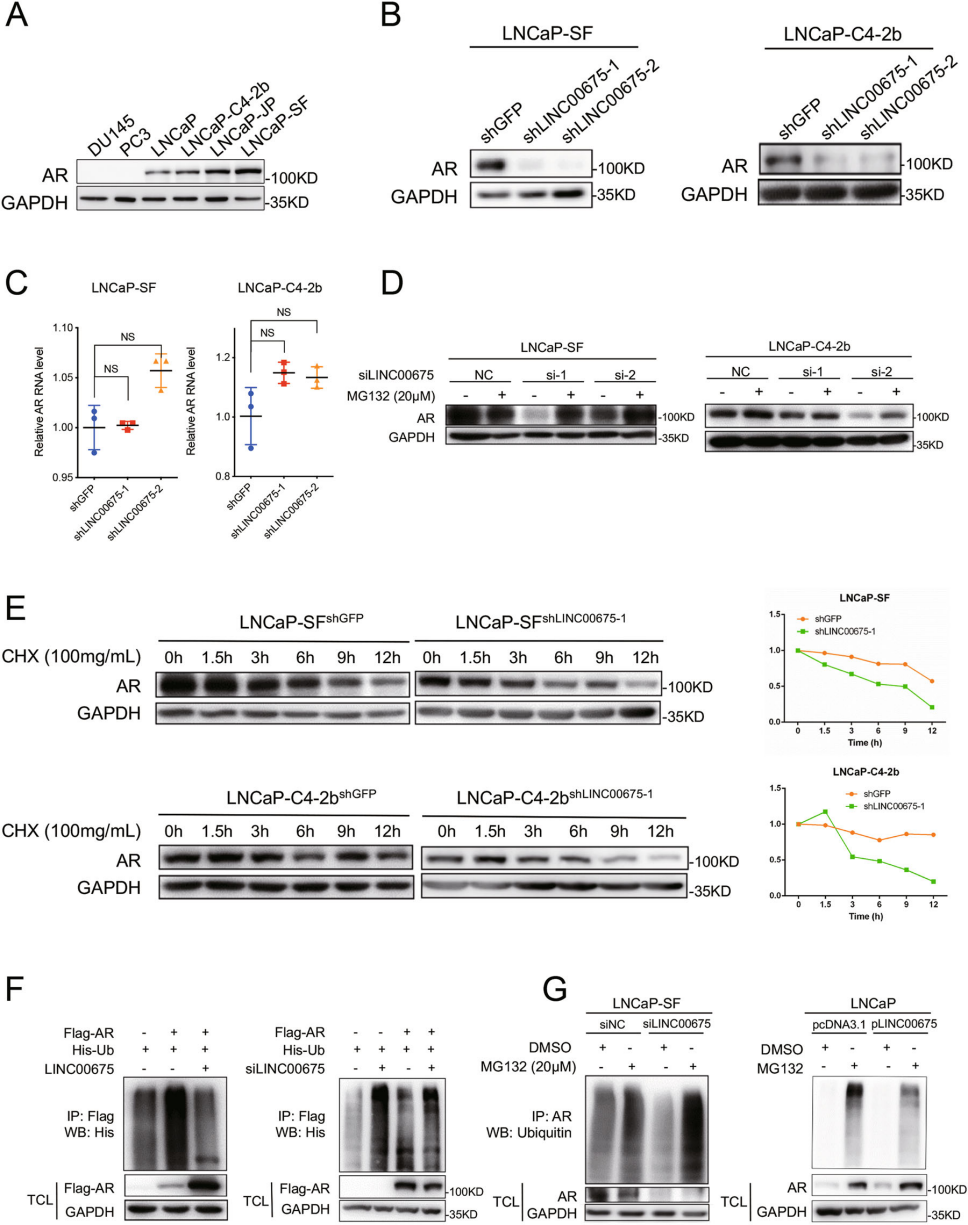
LINC00675 stabilizes androgen receptor (AR) protein by block its ubiquitination. **a** AR expression levels in different prostate cancer (PCa) cell lines. **b** AR protein expression was downregulated in LINC00675-depleted LNCaP-SF and LNCaP-C4-2b cells. **c** AR mRNA expression was not changed in LINC00675-depleted LNCaP-SF and LNCaP-C4-2b cells. Data are represented as mean ± SD. *n* = 3. **d** LNCaP-SF and LNCaP-C4-2b cells were transfected with siRNA to knock down LINC00675. After 24 h, the cells were treated with 20 μM MG132 for 6–8 h and harvested. Western blot showed that AR expression was rescued after treatment. **e** LINC00675-depleted LNCaP-SF and LNCaP-C4-2b cells were treated with 100 mg/ml CHX and harvested at different time points as indicated. AR’s half-life is shortened after LINC00675 depletion. CHX cycloheximide. **f**, **g** LINC00675 blocks ubiquitination of AR confirmed by exogenous and endogenous Co-IP assay, detected by western blot. **f** 293T cells were transfected with Flag-AR, His-Ub, pLINC00675 plasmids, or siLINC00675 as indicated. **g** LNCaP-SF and LNCaP cells were transfected with siLINC00675 or pLINC00675 and relative controls, and then treated with MG132 (20 μM) for 6–8 h before harvest. TCL total cell lysate.

Next, using Gene Set Enrichment Analysis (GSEA) with the above PCa cell lines RNA-seq data, we found that the AR signaling pathway was upregulated (Supplementary Fig. S3A). Also, in our RNA-scope tissue samples, we further conducted consecutive immunohistochemistry of AR and found that LINC00675 and AR were co-expressed (Supplementary Fig. S3B), which suggests that LINC00675 might mediate the AR signaling pathway. Expression levels of AR protein in LNCaP-SF and LNCaP-C4-2b cells were higher than their respective controls (Fig. 3a). Notably, while RT-qPCR showed AR mRNA expression levels were not changed (Fig. 3c), AR expression levels were nearly absent in LINC00675 knockdown LNCaP-SF and LNCaP-C4-2b as observed in western blot assays (Fig. 3b), indicating that LINC00675 is responsible for regulating AR expression at post-translational level, but not in mRNA expression levels.

The ubiquitin–proteasome-degradation pathway is one of the main pathways of intracellular protein degradation^14^. In this regard, we suppose AR expression levels may be regulated via ubiquitination. To test the possibility, LNCaP-SF and LNCaP-C4-2b cells with LINC00675 knockdown were firstly treated with MG132 to inhibit proteasome activity, and we found that AR expression was markedly restored compared with the controls (Fig. 3d). Then we added cycloheximide (CHX) to inhibit protein synthesis to observe whether AR expression was affected by LINC00675. As expected, degradation of AR protein was faster in LNCaP-SF and LNCaP-C4-2b cells with the absence of LINC00675 cells than with the presence of LINC00675, which was further confirmed by quantification analyses (Fig. 3e). These data suggest that LINC00675 knockdown leads to proteasome-mediated AR degradation rather than inhibiting AR translation. Moreover, co-immunoprecipitation (IP) assays were performed to examine whether LINC00675 affects AR ubiquitination. Exogenous IP was conducted in 293T cells with Flag-AR, His-ubiquitin, LINC00675 plasmids, or siRNA co-transfected. Western blot showed that AR poly-ubiquitination was reduced when LINC00675 was overexpressed, whereas AR protein was robustly poly-ubiquitinated when LINC00675 was silenced (Fig. 3f). Notably, endogenous IP was performed using cell lysate from LINC00675-silenced LNCaP-SF cells and LINC00675-overexpressed LNCaP cells treated with 20 μM MG132. In LINC00675-silenced LNCaP-SF cells, AR ubiquitination was highly increased, and total AR protein expression was greatly repressed compared with the respective controls; whereas AR ubiquitination was repressed and total AR protein expression was enhanced in LINC00675 overexpressed LNCaP cells (Fig. 3g).

Above all, these data demonstrate that LINC00675 regulates AR–ubiquitin complex through ubiquitin-dependent proteasomal degradation.

### MDM2 works as an E3 ligase in AR ubiquitination

**Fig. 4:**
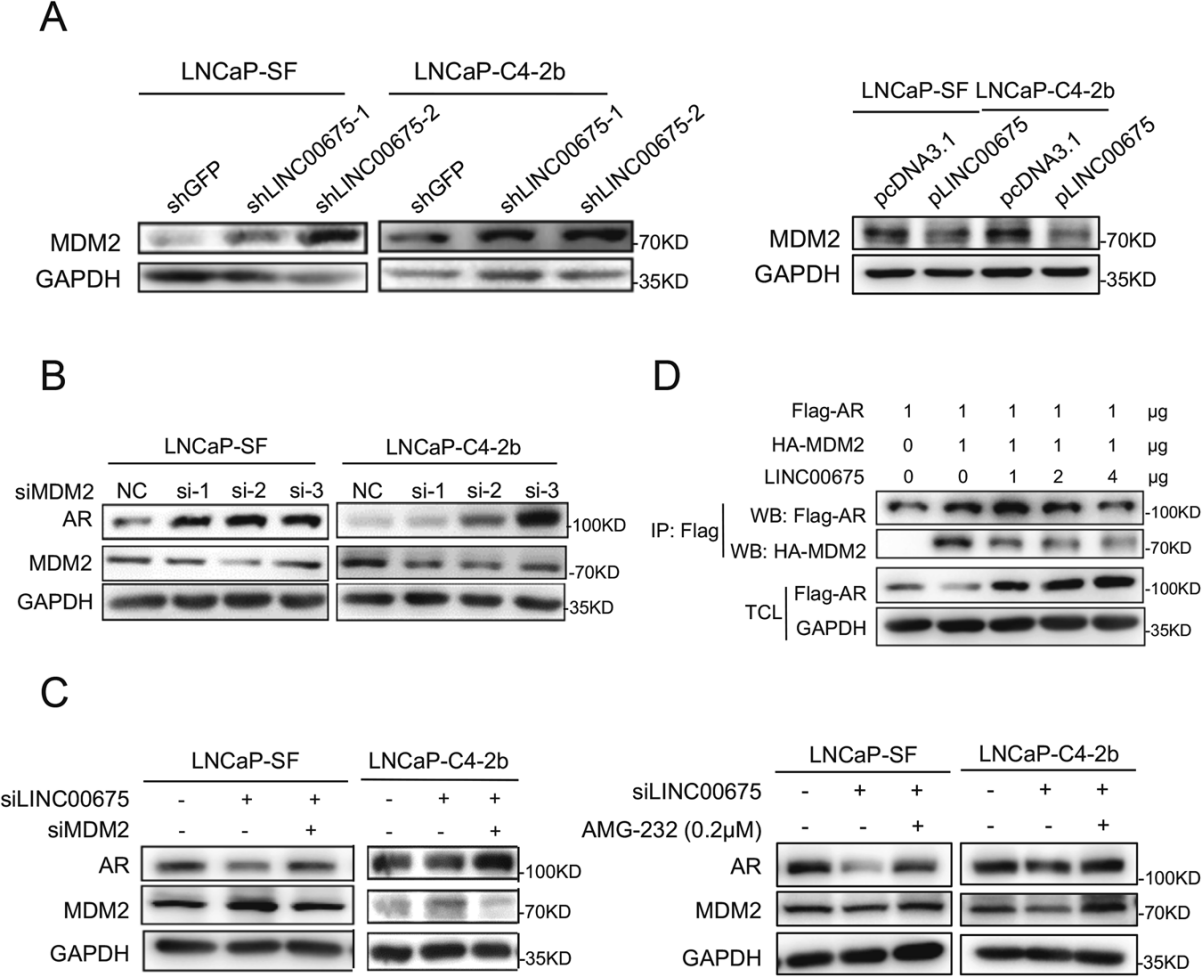
LINC00675 regulates MDM2–androgen receptor (AR) interaction. **a** MDM2 protein expression levels in LNCaP-SF and LNCaP-C4-2b cells with LINC00675 knockdown or overexpression. **b** AR protein expression levels were upregulated after MDMD2 knockdown. **c** AR expression was repressed by knockdown of LINC00675 and restored by knockdown of MDM2. LNCaP-SF and LNCaP-C4-2b cells were transfected with siLINC00675 and siMDM2, or treated with AMG-232 24 h later. The cells were harvested after 24–48 h of treatment. **d** LINC00675 regulates MDM2–AR interaction. 293T cells were transfected with Flag-AR, HA-MDM2, and different concentration of pLINC00675 followed by Co-IP assay and western blot using antibodies as indicated.

Previous studies showed that E3 ubiquitin ligase–MDM2 could bind to the N-terminal domain (NTD) of AR to promote AR–ubiquitin interaction and AR ubiquitination^14^. First, co-IP of AR and MDM2 was conducted to verify the interaction between AR and MDM2 (Supplementary Fig. S3C, D). Meanwhile, expressions of MDM2 in LINC00675-silenced LNCaP-SF and LNCaP-C4-2b cells were detected using western blot, and we found that MDM2 expression levels were upregulated (Fig. 4a). In contrast, MDM2 expression levels in LNCaP-SF and LNCaP-C4-2b cells with overexpressed LINC00675 were decreased (Fig. 4a). In addition, we knock down MDM2 expressions in LNCaP-SF and LNCaP-C4-2b cells with siRNAs, and found that AR protein expression levels were upregulated (Fig. 4b). So we assumed that LINC00675 might affect the interaction between AR and MDM2, thus repressing AR ubiquitination. To test this assumption, knockdown of LINC00675 and MDM2 was performed to reduce expressions of LINC00675 and MDM2 in LNCaP-SF and LNCaP-C4-2b cells. Western blot showed that knockdown of LINC00675 resulted in AR downregulation, and further knockdown of MDM2 lead to restoration of AR protein expression. Similar results were observed when siRNA of MDM2 was replaced by treatment of an MDM2 inhibitor AMG-232 (Fig. 4c).

Moreover, Flag-AR, HA-MDM2, and different concentrations of LINC00675 were co-transfected into the 293T cells. Exogenous co-IP showed that LINC00675 could repress the interaction between AR and MDM2, and the repression effects were dose-dependent (Fig. 4d). In summary, LINC00675 could bind AR to disrupt the interaction between AR and MDM2, thus decreasing AR ubiquitination and protein degradation.

### LINC00675 physically interacts with AR

**Fig. 5:**
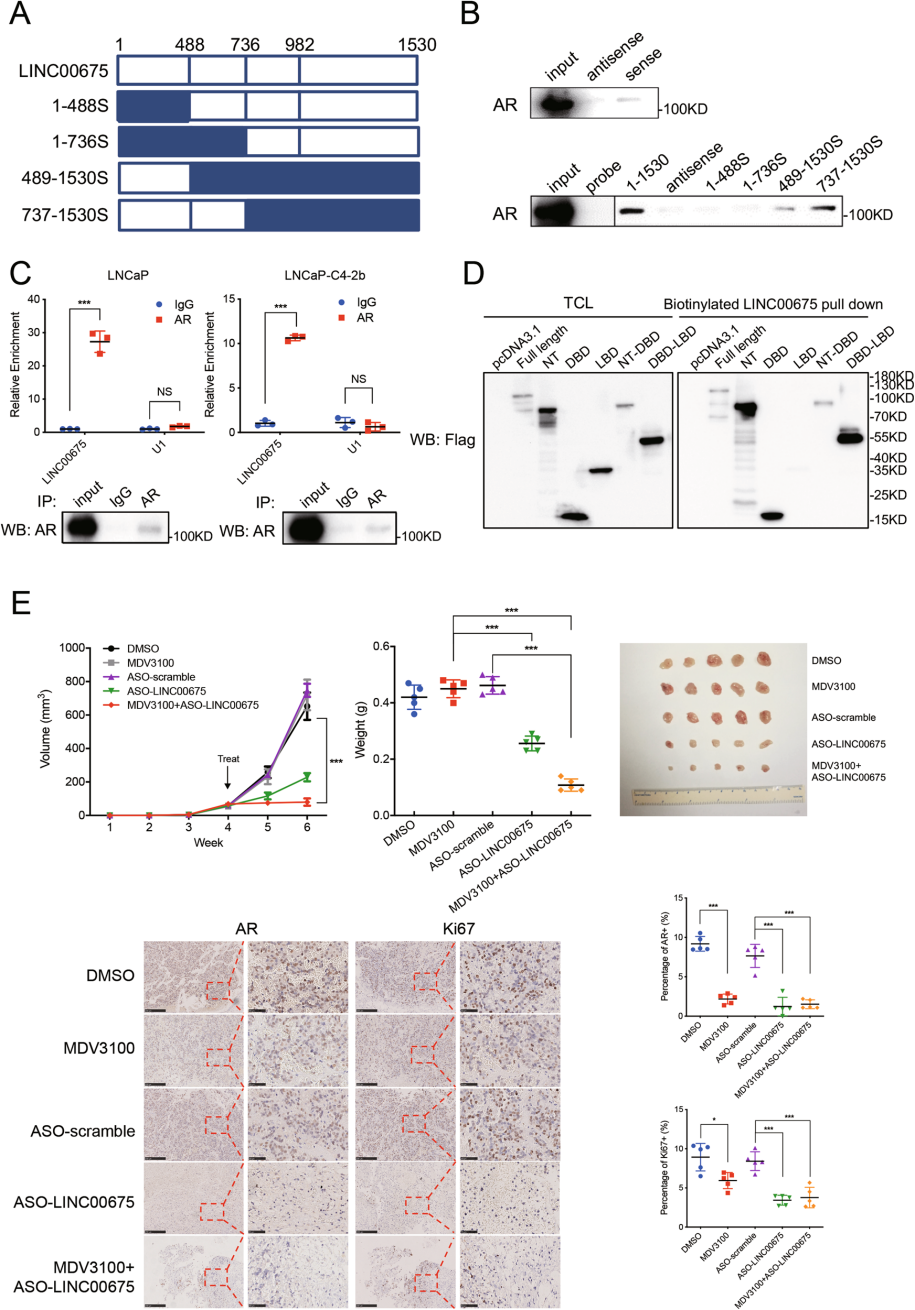
LINC00675 physically interacts with androgen receptor (AR). **a** Schematic diagram outlining the full-length and four different truncated LINC00675 transcripts. **b** RNA pull-down assay showed that LINC00675 binds to AR in its 737–1530 nt area. **c** LINC00675 directly interacts with AR protein by RIP assay. Data are represented as mean ± SD. *n* = 3. **d** LINC00675 binds to the NT and DBD of AR protein. 293T cells were transfected with pcDNA3.1-AR or pcDNA3.1-AR-truncated plasmids, and harvested after 48 h. RNA pull-down assay and western blot were performed to detect binding of LINC00675 and AR. NT N-terminal, DBD DNA-binding domain, LBD ligand-binding domain. **e** Silencing LINC00675 suppresses tumor growth in vivo and benefits androgen-deprivation- insensitive models. The dissected tumors show that ASO-targeting LINC00675 reduces tumor weight and mass, and reverses MDV3100 resistance in LNCaP-C4-2b-ENZ cells. Representative images of AR and Ki67 staining of the xenograft tumors indicate that targeting LINC00675 suppresses cell proliferation in vivo by modulating AR expression. Semi-quantitative of AR and Ki67 IHC was analyzed using ImageJ. Data are represented as mean ± SD. *n* = 5. Scale bar represents 50 μm. ***P < 0.001. ASO antisense oligonucleotides.

To determine specific areas of LINC00675 that could bind to AR, we constructed four truncated LINC00675, including 1–488S, 1–736S, 489–1530S, and 737–1530S as shown in Fig. 5a. We conducted RNA pull-down assay of full-length LINC00675, its antisense transcript, and truncated transcripts after biotin labeling. Western blot was conducted to determine whether AR protein could bind to certain fragments of LINC00675. As shown in Fig. 5b, AR protein could bind to the full-length LINC00675 and 737–1530 nt of LINC00675, while there was no sign of AR protein antisense LINC00675 and 5′-region of LINC00675 pulldown. Furthermore, we conducted anti-AR RIP to confirm the enrichment of AR on LINC00675 transcripts in LNCaP and LNCaP-C4-2b cells (Fig. 5c). As shown in Fig. 5d, total cell lysates (TCL) of 293T cells transfected with AR-Flag or truncated AR-Flag plasmids and biotinylated LINC00675 were incubated and precipitated for western blot analysis. Results show that LINC00675 binds to the NTD of AR where MDM2 binds. Therefore, we suppose that LINC00675 might bind to the NTD of AR to block binding area between AR and MDM2, and induce separation of AR and MDM2, thus resulting in lower level of AR ubiquitination and enhancing protein stabilization. Using RT-qPCR and western blot, we investigated the expression levels of some representative AR target genes, which showed that TMPRSS2 and FKBP5 were upregulated in LINC00675-overexpressed LNCaP-SF and LNCaP-C4-2b cells, while PSA was not significantly changed compared with respective controls (Supplementary Fig. S3E).

To evaluate whether LINC00675 silencing would promote ADT, we conducted in vivo subcutaneous xenograft study with antisense oligonucleotides (ASO) targeting LINC00675 with or without enzalutamide (Supplementary Fig. S3F). Nude mice were xenografted with LNCaP-C4-2b-ENZ cells and treated with scramble or LINC00675 ASO and with DMSO or enzalutamide. The tumor growth was significantly repressed in the ASO-LINC00675 group and combination group than the control group, enzalutamide group, and ASO-scramble group. Decreased levels of AR, Ki67 were confirmed by immunohistochemistry in ASO-LINC00675 and combination group (Fig. 5e).

### LINC00675 interacts with GATA2 mRNA and activates the AR signaling pathway

**Fig. 6:**
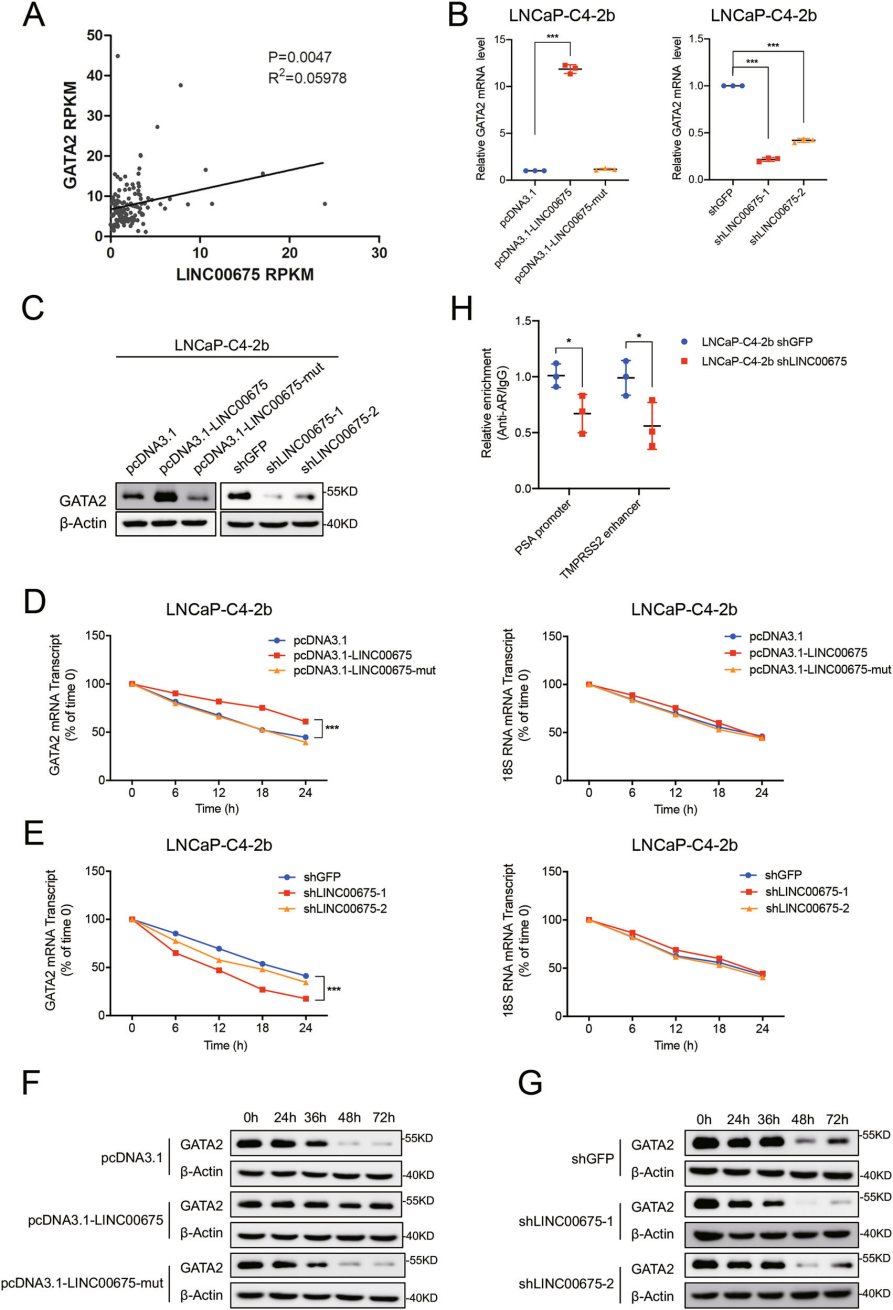
LINC00675 interacts with GATA2 mRNA and activates the androgen receptor (AR) signaling pathway. **a** The correlation between the transcription levels of LINC00675 and GATA2 in primary prostate cancer tissues was determined in our RNA-seq data. Data were analyzed by Spearman’s correlation analysis. **b**, **c** LINC00675 interacts with GATA2 mRNA in LNCaP-C4-2b cells. Data are represented as mean ± SD. *n* = 3. **d**–**g** LINC00675 affects the half-life of GATA2 mRNA. LNCaP-C4-2b cells were treated with α-amanitin (50 μM) to block new RNA synthesis and harvested at different time points as indicated, and then the stability of GATA2 and 18sRNA mRNA was measured by RT-qPCR (**d**, **e**), and the stability of GATA2 and β-actin protein was measured by western blot (**f**, **g**). **h** Knockdown of LINC00675 decreases AR binding to PSA promoter and TMPRSS2 enhancer.

We examined the expression levels of previously reported PCa-related TFs and AR co-regulators in our cohort and found that GATA2 transcript was positively correlated with LINC00675 (Fig. 6a). GATA2 has been reported to play a key role in driving PCa progression by increasing AR binding and activity^23^. Many lncRNAs have been reported to interact with and increase the stability of mRNA^24^. We thus reasoned that LINC00675 might promote progression through binding to GATA2 mRNA and activation of the AR signaling axis. Using BLAST, we identified high complementary regions between LINC00675 and GATA2 mRNA (Supplementary Fig. S4A). We then mutated binding sites on LINC00675. To validate the interaction of LINC00675 with GATA2, we performed RT-qPCR and western blot to test GATA2 expression levels in LNCaP and LNCaP-C4-2b cells transfected with LINC00675, GATA2-binding sites mutated LINC00675, and their relative controls (Fig. 6b, c; Supplementary Fig. S4B, C). These results suggested that LINC00675 interacts with GATA2 mRNA. To test whether LINC00675 regulates the stability of GATA2 mRNA, we treated PCa cells with α-amanitin to block new RNA synthesis and then measured the decrease of GATA2 and 18sRNA. The overexpression of LINC00675, but not LINC00675-mut extended the half-life of GATA2 mRNA, and conversely, the depletion of LINC00675 condensed the half-life of GATA mRNA (Fig. 6d–g; Supplementary Fig. S4D, E). Alterations of LINC00675 expression decreased the expression levels of AR and GATA2, as shown in Figs. 3b, 6c, we also conducted ChIP using anti-AR to perform PCR in the PSA promoter and TMPRSS2 enhancer region. The results suggest that knockdown of LINC00675 highly decreased AR binding to the PSA promoter and TMPRSS2 enhancer (Fig. 6h). Collectively, these data demonstrate that LINC00675 increases the stability of GATA2 mRNA through binding to the GATA2 transcript.

## Discussion

LncRNAs participate in cancer development and progression via diverse mechanisms of regulating transcriptional, translational, and protein function. LncRNAs could act as a transcriptional activator, repressor, guide, or scaffold for chromatin-modification complex^25,26^. In addition to transcriptional regulation by epigenetic changes, lncRNAs are also known to be involved in RNA processing as an RNA editing/splicing regulator, miRNA harbor, miRNA sponges, miRNA blocker, RNA degradation regulator, and translational efficiency regulator^27,28^. Our expression profiles of PCa cell lines ranging from androgen-dependent to androgen-independent identified differentially expressed lncRNAs.

The AR signaling axis is critical in PCa development and progression, and androgen deprivation represents first-line treatment for late-stage PCa patients^2,29^. AR is the most commonly altered gene in CRPC, leading to reactivation of AR signaling in CRPC^30^. Even with the introduction of abiraterone and enzalutamide for metastatic CRPC treatment, patients benefited for a limited response time and cancer progression inevitably. Normally, AR exists in the cytoplasm as an inactivated complex with heat-shock proteins (HSP), while E3 ubiquitin ligase–MDM2 recruits AR via binding to its NTD and mediates ubiquitination degradation of the protein^31^. HOTAIR (HOX transcript antisense RNA) was reported as an androgen-repressed lncRNA that could bind AR protein to protect it from ubiquitin-mediated degradation^14^. The study showed that HOTAIR could bind to AR protein to block its interaction with MDM2. Zhang et al. reported that ARLNC1 could bind to AR mRNA directly and stabilize AR transcript via RNA–RNA interaction^32^. In this study, we found that LINC00675 could directly bind to NTD of AR in specific regions as a protector to block the binding area between AR and MDM2, and thus decrease ubiquitination levels of AR to increase the stability of AR protein.

As a mediator of AR signaling, it has been reported that GATA2 plays a critical role in sustaining both AR-dependent and -independent PCa progression^33,34^. GATA2 could bind to the upstream promoter region of AR and facilitate AR expression; in addition, GATA2 recruit p300 for H3K27 acetylation to create an active and accessible chromatin environment^34^. GATA2 expression levels are upregulated in CRPC tumor samples than primary cancer samples^33^, and GATA2 expression levels increase throughout the transition from androgen-dependent status to castration-resistant PCa xenograft mouse model^35^. Notably, our findings demonstrate that LINC00675 could bind to GATA2 transcript in addition to AR protein. On one hand, LINC00675 directly binds to the NTD of AR protein to prevent its interaction with MDM2 via the same domain, thus LINC00675 inhibited AR ubiquitination-mediated degradation and increased AR protein stability via a competitive mechanism. On the other hand, LINC00675 promotes AR signaling by binding GATA2 mRNA, increasing GATA2 mRNA stability, causing increased GATA2 co-activation of AR and promoting PCa progression.

LncRNAs could be degraded by siRNAs or ASOs and targeted by small-molecular inhibitors to disrupt downstream pathways^36^. Targeting the MALAT1/AR-V7 axis with the drug ASC-J9 might be effective in patients insensitive to enzalutamide treatment^37^. Also, the drug MS351 as a PRC1 complex inhibitor could disrupt the chromatin-modifying complexes between PRC2 and HOTAIR^38^. In this study, we treated subcutaneous xenografts models with enzalutamide and ASO-targeting-LINC00675 in vivo and found that targeting LINC00675 would benefit androgen-deprivation insensitive models, which suggest that the LINC00675/AR axis may be a promising drug target in ADT-insensitive cases.

Although we have explored the role of LINC00675 in AR signaling during CRPC, the molecular mechanism leading to LINC00675 upregulation in CRPC remains to be fully illustrated. In addition, whether the physical interaction between LINC00675 and AR protein, and binding of LINC00675 and GATA2 mRNA transcript occurs with additional RNA-binding protein need to be illustrated.

**Fig. 7:**
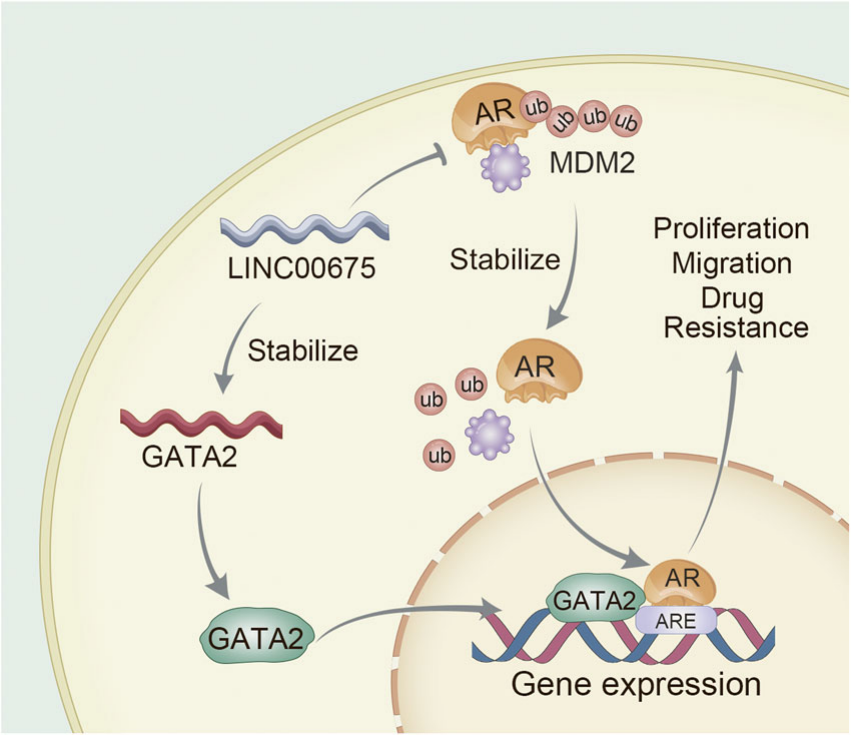
LINC00675 promotes castration resistance. Schematic diagrams demonstrating the mechanism of the LINC0067/MDM2/AR/GATA2 axis signaling pathway.

In summary, it is our novel discovery that LINC00675 promotes castration resistance by binding AR, blocking its recruitment with MDM2 and protecting it from ubiquitin-mediated degradation. LINC00675 could bind to GATA2 mRNA and enhance its stability, thus GATA2 protein could act as a co-activator of AR (Fig. 7). Our findings suggest that the LINC00675/MDM2/GATA2/AR signaling axis is a potential therapeutic target for CRPC.

## Supplementary information

supplementary materials

Supplementary Figure S1

Supplementary Figure S2

Supplementary Figure S3

Supplementary Figure S4
