# Supplementary material for: LINC00675 activates androgen receptor axis signaling pathway to promote castration-resistant prostate cancer progression
Source: Cell Death Dis. 2020 Aug 15;11(8):638. doi: 10.1038/s41419-020-02856-5 (PMC7429955; doi:10.1038/s41419-020-02856-5)
Supplement: Supplementary file 1 — supplementary materials [file 41419_2020_2856_MOESM1_ESM.docx]

**Supplementary Materials**

Yao M, et al. LINC00675 activates androgen receptor axis signaling pathway to promote castration resistant prostate cancer progression.

**Supplementary Figure Legends**

**Figure S1 Long non-coding RNA LINC00675 suppresses cell growth and highly expresses in CRPC**

(A) Loss of function assay of 7 different lncRNAs in LNCaP-SF cell line with two different siRNAs.

(B) Protein coding potential of LINC00675 was performed by UCSC, and txCdsPredict score showed no potential of protein coding.

(C) LINC00675 expression and localization on enzalutamide sensitive LNCaP-C4-2b and enzalutamide resistant LNCaP-C4-2b-ENZ cells. LINC00675 expression was higher in cytoplasma than nucleus, and compared with enzalutamide sensitive LNCaP-C4-2b cells, LINC00675 expression was higher in LNCaP-C4-2b-ENZ cells.

(D) LINC00675 expression was higher in CRPC tissues than primary PCa tissues of patients, examined using RT-qPCR.

*: *P*<0.05; **: *P*<0.01.

**Figure S2 Knockdown of LINC00675 reverses drug resistance and represses PCa progression**

(A) LINC00675 knockdown in LNCaP-C4-2b-ENZ cells reversed enzalutamide resistance. LNCaP-C4-2b-ENZ cells were treated with 20 μM MDV3100.

(B) Alterations of LINC00675 expression affect EMT signaling pathway.

(C) Representative images of subcutaneous mice xenografts.

(D) LINC00675 depletion in LNCaP-C4-2b cells suppressed tumor growth *in vivo*. Tumor volumes and weights were measured. RT-qPCR showed LINC00675 expression levels were downregulated. Data are represented as mean±SD. n=12

(E) AR expression was markedly repressed in xenograft tumors after LINC00675 knockdown. Representative images of AR and Ki67 staining of the xenograft tumors indicate LINC00675 knockdown suppresses cell proliferation *in vivo*. Semi-quantitative of AR and Ki67 IHC was analyzed using ImageJ. Data are represented as mean±SD. n=5.

**Figure S3 LINC00675 co-expresses with AR and MDM2 directly interacts with AR.**

(A) Gene Set Enrichment Analysis (GSEA) in the RNA-seq data revealed that AR signaling pathway was up-regulated.

(B) LINC00675 and AR were co-expressed. IHC of AR and RNA-scope of LINC00675 were performed in consecutive tissue samples. Scale bar: 100 μm.

(C-D) Interaction between AR and MDM2 verified by co-IP.

(E) Expression levels of some AR target genes were up-regulated in LINC00675 expressed PCa cells.

(F) ASO effectively decreased LINC00675 expression in mice model. Data are represented as mean±SD. n=3

**Figure S4 LINC00675 stabilizes GATA2 mRNA in LNCaP cells.**

(A) The predicting binding sites of LINC00675 and GATA2 mRNA transcripts.

(B-C) LINC00675 interacts with GATA2 mRNA in LNCaP cells. Data are represented as mean. n=3

(D-E) LINC00675 affects the half-life of GATA2 mRNA in LNCaP cells.

**Supplementary methods**

**RNA extraction, RT-PCR and RT-qPCR**

Total RNA was isolated using TRIzol^TM^ Reagent (15596018, Thermo Fisher Scientific, MA, USA) according to the protocol provided by the manufacturer. Total RNA of 0.5-1 μg was used as templates for reverse transcription using PrimeScriptRT^TM^ Master Mix (RR036A, TAKARA, Japan). Quantitative real-time PCR (RT-qPCR) was conducted using TB Green^TM^ Premix Ex Taq II (RR820A, TAKARA, Japan) according to the manufacturer’s protocol. The primer sequences for the study are listed in the supplementary materials.

**Isolation of cytoplasmic and nuclear RNA**

Cytoplasmic and nuclear RNA were isolated and purified using the NE-PER™ Nuclear and Cytoplasmic Extraction Reagents (7883, Thermo Fisher Scientific, MA, USA) according to the manufacturer’s instructions. The nuclear and cytoplasmic distributions were evaluated by RT-qPCR.

**Migration assay**

Migration assay was performed using Transwell chamber inserted in a 24-well plate. 5×10^4^ cells suspended in 100μl serum-free culture medium were added to the upper chamber. The lower chamber was filled with 500μl normal culture medium. The cells were incubated for 12-24h in a 37 °C, 5% CO_2_ atmosphere. After this, cells on the upper surface of the chamber were wiped away, and cells on the lower surface were fixed with 4% paraformaldehyde and stained with 0.1% crystal violet at room temperature for 30 min. At lease 5 random fields were counted under an optical microscope.

**Colony formation assay**

Cells were resuspended and 1×10^3^ cells were seeded into 6-well plates. The culture medium was added every 3 days until the colonies were visible. After 14 days, the cells were fixed with 4% paraformaldehyde and stained with 0.1% crystal violet at room temperature for 30 min. The colonies were counted using GelCount (Oxford Optronix, UK).

**RNA-immunoprecipitation (RIP)**

RIP assays were conducted using the EZ-Magna RIP RNA-Binding Protein Immunopreciptation Kit (17-700, Millipore, MA, USA). The AR-based RIP assay was performed according to previous report (Shi, et al. 2019). The RNA fractions precipitated by RIP were analyzed by RT-qPCR. Antibodies of AR (5153, CST, MA, USA; 74272, Abcam, MA, USA) and purified IgG (PP64-KSP, Millipore, MA, USA) were commercially purchased.

**Co-Immunoprecipitation (IP)**

500-1000 μg proteins were incubated with the indicated antibody at 4 °C overnight. Protein A/G PLUS Agarose beads (sc-2003, Santa Cruz Biotechnology, TX, USA) were then added and the mixture was incubated at 4 °C for additional 1-2 h. Beads were washed at least three times with lysis buffer. Bound proteins and 10% input were detected by western blot with antibodies as indicated.

**Fluorescence *in situ* hybridization**

RNA probe labeled with digoxigenin was designed and purchased from QIAGEN (Germany). Probe sequence: AGCACTGGCTGTACGTTCACA. LNCaP-C4-2b and LNCaP-C4-2b-ENZ cells were seeded in glass-bottom culture dishes a day before FISH assay, and the cells were fixed with 4% paraformaldehyde for 30 min at room temperature. Then add 3% BSA/4×SSC buffer to the culture dishes and place the dishes in a 54 °C water bath for 20 min to prepare hybridization. Dilute RNA probe with hybridization buffer (10% DSS/4×SSC) to an appropriate concentration and preheat to 54 °C. Replace 3% BSA/4×SSC buffer with RNA probe/hybridization buffer mix and place the dishes in a 54 °C water bath for 1 h or longer. Replace the buffer with wash buffer I (4×SSC + 0.1% Tween-20) and put the dishes in a 59 °C water bath. Wash it for 5 min, 3 times. Wash buffer II (2×SSC) for 5 min at 59 °C, wash buffer III (1×SSC) for 5 min at 59 °C and 1×PBS for 5 min at room temperature. Afterwards, place 200 μl blocking solution (5% BSA) on the hybridized cells and incubate at room temperature for 1 h. Replace blocking solution with digoxigenin antibody and incubate at 4 °C overnight. Wash the cells with 1×PBS, replace PBS every 5 min for 15 min, and then add fluorescent secondary antibody, incubate at room temperature for 1 h avoiding light. DAPI was add to stain chromosomes before the cells were viewed using confocal microscopy.

**Antibodies**

Ki-67: ab15580, Abcam, MA, USA

AR: 74272, Abcam, MA, USA

GAPDH: 60004-1-Ig, Proteintech, IL, USA

Flag-tag: F1804, Sigma-Aldrich, MO, USA

Ub: 3933, Cell Signaling Technology, MA, USA

His-tag: 12698, Cell Signaling Technology, MA, USA

HA-tag: 3724, Cell Signaling Technology, MA, USA

MDM2: ab16895, Abcam, MA, USA

E-cadherin: ab40772, Abcam, MA, USA

N-cadherin: ab76011, Abcam, MA, USA

Vimentin: 5741, Cell Signaling Technology, MA, USA

**Primer sequences**

RP11-267A15.1 forward: 5'-CTGAAGCATGGCATTGAGAA-3'

RP11-267A15.1 reverse: 5'- CTCACTCCTTCATGCGTTGA-3'

LINC00472 forward: 5'- GTGGCCTTAAAACCCCATTT-3'

LINC00472 reverse: 5'- AAATAACGGGGGCTACCATC-3'

RP1-90L14.1 forward: 5'- ATGCTGAACATGGCCTATCC-3'

RP1-90L14.1 reverse: 5'- GTGTCCTCATCCCTTCAGGA-3'

SNORD3A forward: 5'- CCACGAGGAAGAGAGGTAGC-3'

SNORD3A reverse: 5'- CACTCCCCAATACGGAGAGA-3'

RNU12 forward: 5'- AAATAACGATTCGGGGTGAC-3'

RNU12 reverse: 5'- CAGGCATCCCGCAAAGTAG-3'

EGFR-AS1 forward: 5'- AACACCGGTCTTCACCAAAG-3'

EGFR-AS1 reverse: 5'- TGGTTAGCAGTGGCAGTCAG-3'

MIR210HG forward: 5'- GTACGACCATCCCTCCAGAA-3'

MIR210HG reverse: 5'- CTGGAATGCATAGGCCAAGT-3'

NEAT1 forward: 5'- CTTCCTCCCTTTAACTTATCCATTCAC-3'

NEAT1 reverse: 5'-CTCTTCCTCCACCATTACCAACAATAC-3'

AC009410.1 forward: 5'- ATTCAATTTGGCTCCTGCTG-3'

AC009410.1 reverse: 5'- TGGGAGGATGAAGTTGAAGG-3'

RP11-90K6.1 forward: 5'- TCCTTTGCCTGTAGGTGGTC-3'

RP11-90K6.1 reverse: 5'- AGTGGAAGAGCCGTTTCCTT-3'

CTC-537E7.1 forward: 5'- TCATGGAAGGAAGCTGGATT-3'

CTC-537E7.1 reverse: 5'- CCATTTCTGTCAGCCATTGA-3'

RP11-274B21.8 forward: 5'- CATCCTTGCTAAGCGTGTCA-3'

RP11-274B21.8 reverse: 5'- TATTGCCAACAGCAAACAGC-3'

CTD-2589M5.4 forward: 5'- TGCCTGTTCCAGACACTGAG-3'

CTD-2589M5.4 reverse: 5'- ATCTTCCACACATGGCTTCC-3'

RP11-74E22.6 forward: 5'- ATGTGGCTCACGTTCAACAA-3'

RP11-74E22.6 reverse: 5'- CACCAGGAACATACGCCTTT-3'

CTD-2540B15.12 forward: 5'- GCCACAGGCTTCATTTCTTC-3'

CTD-2540B15.12 reverse: 5'- GCTCCCAAGCTTCTGTCATC-3'

RP1-288H2.4 forward: 5'- CTTACCCCAGGAGCAGTGAG-3'

RP1-288H2.4 reverse: 5'- AGCATGCCGTTCAAATTTTC-3'

RP1-288H2.2 forward: 5'- CTTGGGACCTAGCACCATGT-3'

RP1-288H2.2 reverse: 5'- TTGCTGTCCTTGCTTCCTCT-3'

RP11-398J5.1 forward: 5'- AGAGCACCCAACTCCTCTGA-3'

RP11-398J5.1 reverse: 5'- CAGGGAGCTCAGTCTTCACC-3'

DLEU2 forward: 5'- CTCTAACGAATTTGAATGAGGAGC-3'

DLEU2 reverse: 5'- CTACAATGATAATTTGGTCTTACTCTGAGTTAAAT-3'

MIR133A1 forward: 5'- AGCGCAGGAAAACAGTAGGA-3'

MIR133A1 reverse: 5'- CATTGAAGAGGCGATTTGGT-3'

LINC01057 forward: 5'- GATGAACAGGCTCTTGCACA-3'

LINC01057 reverse: 5'- TGCCTTGTCAGCACTTCATC-3'

RP11-371I1.2 forward: 5'- TATGGAGGATCGCTGTTTCC-3'

RP11-371I1.2 reverse: 5'- CTCTGGTTGGGTGACTGGTT-3'

RP11-255C15.4 forward: 5'- TCTTCGCAGCAGTGAACAGT-3'

RP11-255C15.4 reverse: 5'- AAGCAGGAAAAGAAGAGGCTTT-3'

RP11-586D19.1 forward: 5'- TGAGACCCATTGGACACAGA-3'

RP11-586D19.1 reverse: 5'- AGAGGCTCCAGGTGCTATGA-3'

RP11-351E7.1 forward: 5'- TCATCTTTCATGCAGCCTGT-3'

RP11-351E7.1 reverse: 5'- GATATCTGGTGGTGCTCAGGA-3'

RP11-945A11.1 forward: 5'- CACCTTCGGAGAGGTCTGAG-3'

RP11-945A11.1 reverse: 5'- CTATTTGGCCATCTGGTGCT-3'

AP004372.1 forward: 5'- TCCCCAAACTCAATGAGGAC-3'

AP004372.1 reverse: 5'- GAGTCAGGGGCTCAGAGTTG-3'

LINC00675 forward: 5'- ATCTGACATCGCTGCTGTTG-3'

LINC00675 reverse: 5'- GGACTTTGGTGCTTGTGGTT-3'

RP11-176N18.2 forward: 5'- GGTGCGGCATATAATCTCGT-3'

RP11-176N18.2 reverse: 5'- GCGACACAGAAGATGGTTGA-3'

AF131217.1 forward: 5'- CTTCTCAGTGGGGTCTCTGC-3'

AF131217.1 reverse: 5'- GACATTCTGCCTTCCACCAT-3'

CTA-150C2.13 forward: 5'- AGGAGACGCTGGTCTGAAAA-3'

CTA-150C2.13 reverse: 5'- CAGTCGGGGATGACAGAAAT-3'

PCR-LINC00675 forward: 5'- CCGCTCGAGGTGGCTCCAAGAAGCGCCAG-3'

PCR-LINC00675 reverse: 5'- CGGAATTCTTACATAAAAACAAAGGCCAAATC

T-3'

T7-LINC00675-sense forward: 5'- TAATACGACTCACTATAGGGAGAGTGGCT

CCAAGAAGCGCCAG-3'

T7-LINC00675-sense reverse: 5'- TTACATAAAAACAAAGGCCAAATCT-3'

T7-LINC00675part1-sense forward: 5'-TAATACGACTCACTATAGGGAGAGTG

GCTCCAAGAAGCGCCAG-3'

T7-LINC00675part1-sense reverse: 5'- GGCGTGGCTTGCTAGGTGGAAG-3'

T7-LINC00675part2-sense forward: 5'- TAATACGACTCACTATAGGGAGAGTG

GCTCCAAGAAGCGCCAG-3'

T7-LINC00675part2-sense reverse: 5'- AGGCCCTTCATAAACAGCTGGC-3'

T7-LINC00675part3-sense forward: 5'-TAATACGACTCACTATAGGGAGAGGG

ACCAGGTCCCATCTGATGG-3'

T7-LINC00675part3-sense reverse: 5'- AAAGGGTAGATACAGTGGAA -3'

T7-LINC00675part4-sense forward: 5'-TAATACGACTCACTATAGGGAGAGAGT

AGCTAGCAGGTTTTTAT-3'

T7-LINC00675part4-sense reverse: 5'-TTACATAAAAACAAAGGCCAAATCT-3'

MDM2 forward: 5'- TCAATCAGCAGGAATCATCG-3'

MDM2 reverse: 5'- GTGGCGTTTTCTTTGTCGTT-3'

U1 forward: 5'- ATACTTACCTGGCAGGGGAG-3'

U1 reverse: 5'- CAGGGGGAAAGCGCGAACGCA-3'

AR forward: 5'- TCCAAATCACCCCCCAGGAA-3'

AR reverse: 5'- GACATCTGAAAGGGGGCATG-3'

PCR AR forward: 5'- CGGAATTCATGGAAGTGCAGTTAGGGCTG-3'

PCR AR reverse: 5'- CCGCTCGAGTCACTGGGTGTGGAAATAGATGG-3'

PSA forward: 5'- GCCTCTCGTGGCAGGGCAGT-3'

PSA reverse: 5'- CTGAGGGTGAACTTGGGCAC-3'

TMPRSS2 forward: 5'- CAGGAGTGTACGGGAATGTGATGGT-3'

TMPRSS2 reverse: 5'- GATTAGCCGTCTGCCCTCATTTGT-3'

FKBP5 forward: 5'- AGCCAAGGGTGACTTTGAGA-3'

FKBP5 reverse: 5'- TCTGCAGTCTTGCAGCCTTA-3'
