# Supplementary figures and images for: LINC00675 activates androgen receptor axis signaling pathway to promote castration-resistant prostate cancer progression
Source: Cell Death Dis. 2020 Aug 15;11(8):638. doi: 10.1038/s41419-020-02856-5 (PMC7429955; doi:10.1038/s41419-020-02856-5)

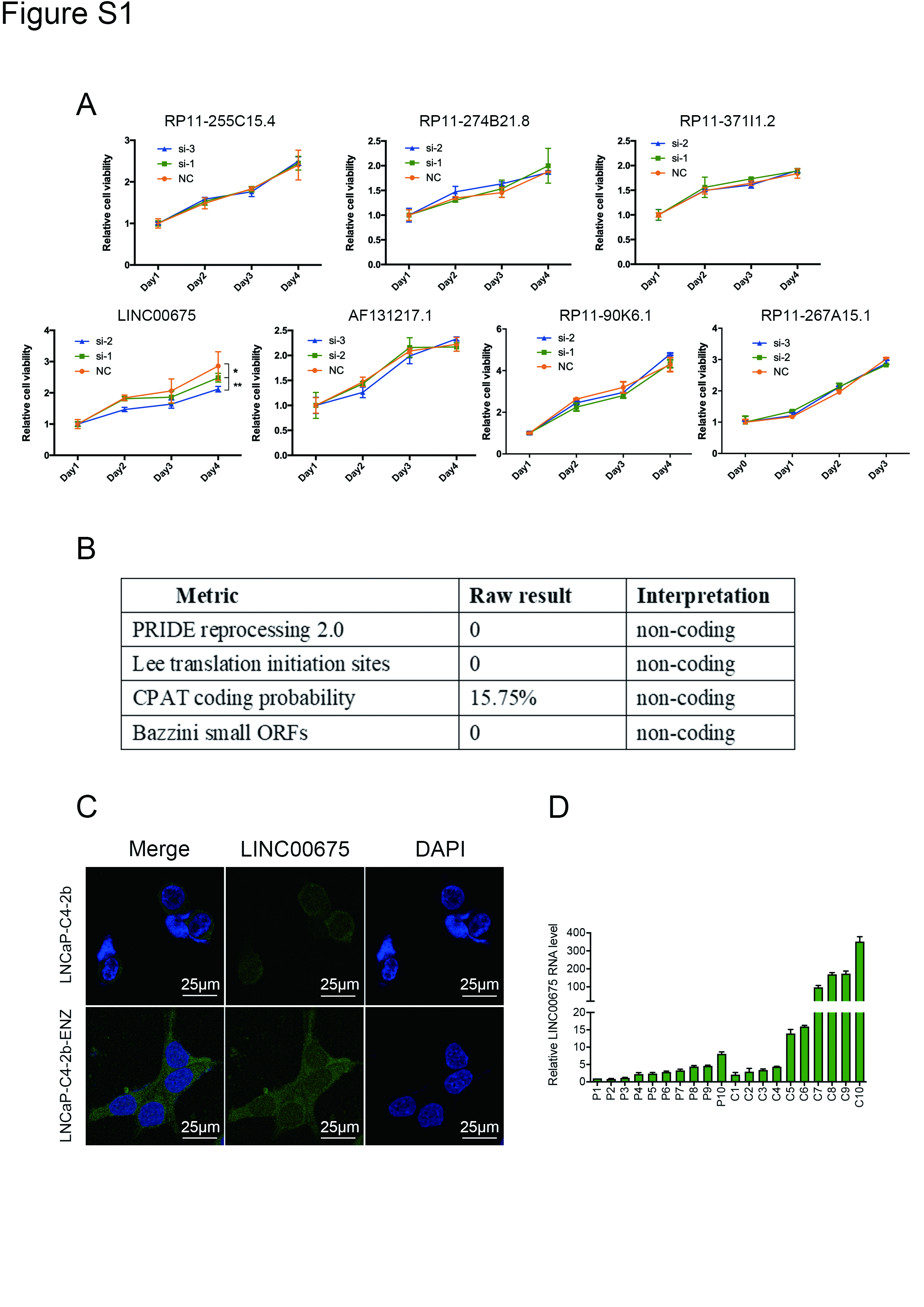

Supplement: Supplementary file 2 — Supplementary Figure S1 [file 41419_2020_2856_MOESM2_ESM.tif]

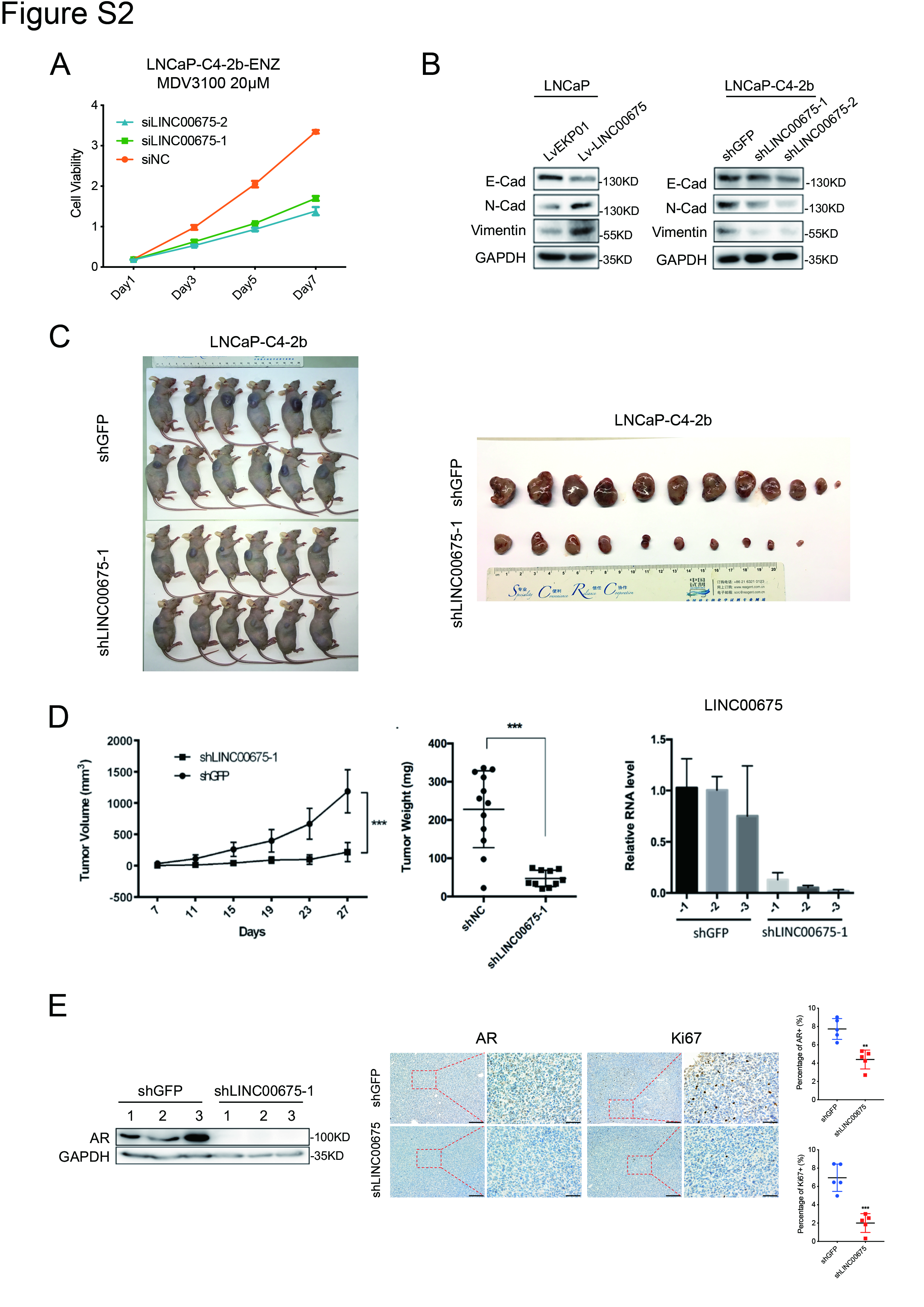

Supplement: Supplementary file 3 — Supplementary Figure S2 [file 41419_2020_2856_MOESM3_ESM.tif]

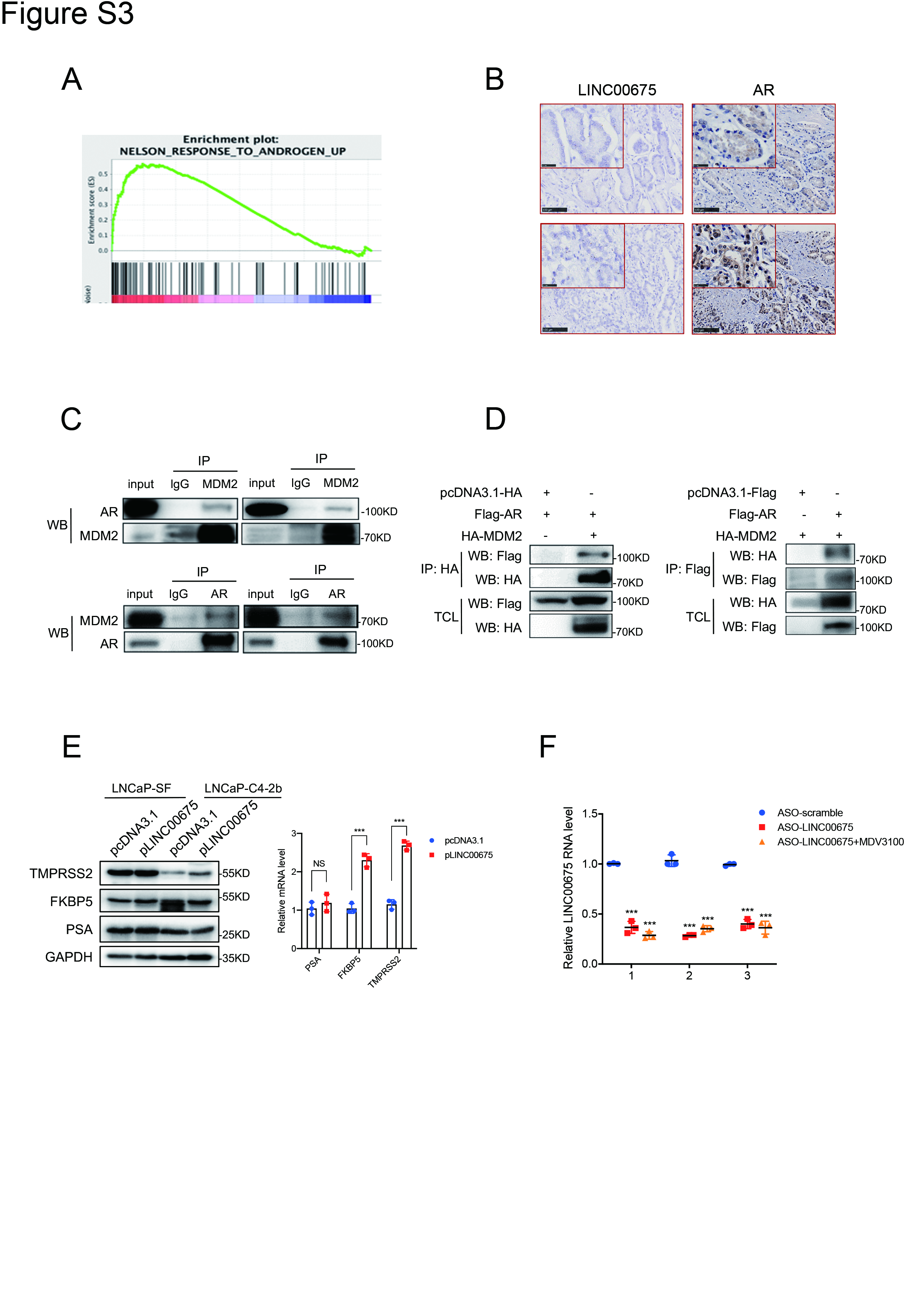

Supplement: Supplementary file 4 — Supplementary Figure S3 [file 41419_2020_2856_MOESM4_ESM.tif]

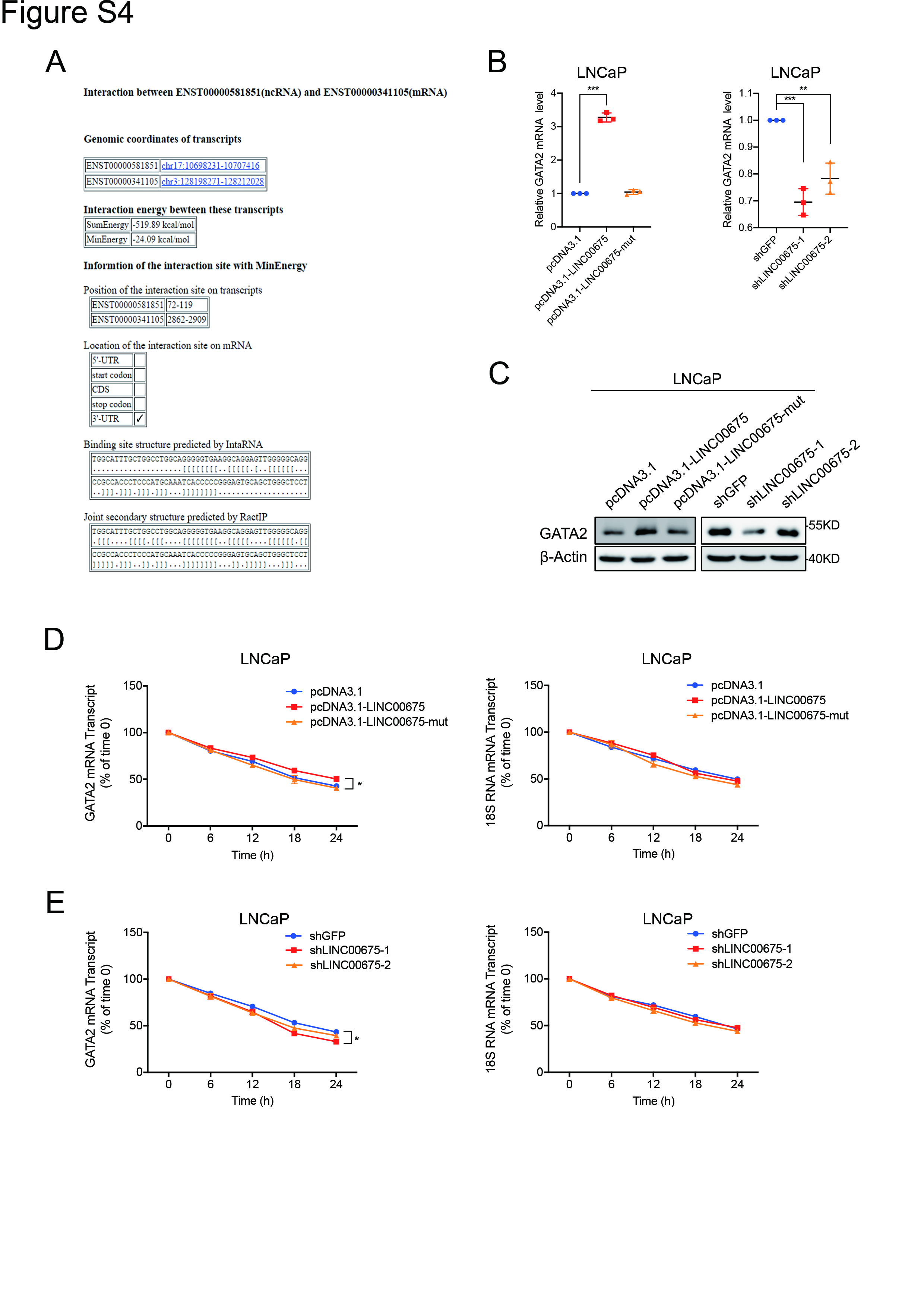

Supplement: Supplementary file 5 — Supplementary Figure S4 [file 41419_2020_2856_MOESM5_ESM.tif]
